# Design of a Computer-Based Legal Information Retrieval System

**DOI:** 10.1155/2022/6942773

**Published:** 2022-05-12

**Authors:** Feng Wu, Yanting Ji, Wenping Shi

**Affiliations:** ^1^Criminal Justice School, Zhongnan University of Economics and Law, Wuhan, China; ^2^International Business School, Sichuan International Studies University, Chongqing 400031, China

## Abstract

In today's society, people's lives are increasingly inseparable from computer information. Due to the continuous improvement of technology and the rapid development of internet technology, the network environment is becoming more and more complex, which makes it easy to cause loopholes in the information retrieval system when people use the network. Therefore, it is especially important to search for legal knowledge by computer. In order to adapt to this change and demand, we need a retrieval system to provide the corresponding search function, legal information content, and management and other services, so as to achieve the purpose of computer legal information retrieval. The legal information retrieval system is computer based, draws conclusions from the analysis of relevant data, and then applies them to judicial trial cases, criminal investigations, and other fields to provide a reference for relevant legal issues. The system is designed to combine computer technology with a criminal investigation and other fields, and then analyze the data to draw the corresponding conclusions. The retrieval algorithms used are mainly image and content retrieval algorithms, and image retrieval algorithms mainly use image segmentation technology, while content retrieval algorithms mainly use the cuckoo algorithm. At present, the information construction and economic and social development in China have become one of the issues of common concern and need to be solved by all countries in the world. The study of the legal information retrieval system is of great importance in the construction of information technology and the development of economic society.

## 1. Introduction

Legal information retrieval is a new method developed on the basis of the development of computer technology to solve the current complex and changing social environment in order to improve the standard and quality of people's lives. With the steady pace of China's economic construction and the continuous improvement of network information technology, this makes the legal information system more and more developed and mature and widely concerned and applied [1]. The development of computers has entered a new era, in which people also put forward higher requirements for information retrieval technology. At present, there are many systems developed in the world about network management, electronic communication, and other fields, which can bring great economic benefits and environmental improvement value to the society, and are developed with data processing as the core content [2, 3]. For the laws and regulations, the procedure has important significance: first, it can effectively protect the legitimate rights and interests of the rights holders so that they can effectively seek help in a timely manner when their legitimate rights and interests are infringed. Second, it can play a preventive role against infringement and safeguard the relevant national interests from damage so that laws and regulations can play their due role in social life. Third, the program can make the search system more perfect and improve the quality of legal information search, so as to promote the process of informationization in China [4].

In this era of information technology, computer technology has widely penetrated into all areas of our life, especially the internet, mobile communication, and other new media. The legal information retrieval system as one of the important components has also received the attention of people from all walks of life, and its research and use have become a popular topic. A computerized legal information retrieval system is designed to solve people's disputes in practice, and its purpose is to provide timely, effective, accurate, and reliable evidence for relevant departments [5]. Under the accelerated development and perfection of the construction of the “Rule of Law China,” the degree of legalization in China has gradually deepened, citizens' awareness of relevant knowledge has gradually strengthened, the development of the legal information retrieval system has been constantly improved, and its significance and value have become increasingly prominent. In such an information era, people are more and more aware of intellectual property protection, which makes it more and more urgent and necessary for us to analyze the potential commercial value of computer technology [6, 7]. In this study, we focus on the legal information retrieval of computer retrieval system, in which we mainly use an image retrieval algorithm and cuckoo-based content retrieval algorithm to design the system.

In the functional design of a legal information retrieval system, the key is how to realize the classification of contents into several categories and distinguish the relationship between different types and categories through the division of rules. The legal image analysis method developed based on computer technology is proposed for the traditional semantic features and text-level semantic characteristics. It has two significant advantages: first, it can directly use database management and other related operations. Second, it can manage the retrieval process effectively. Based on the construction of a computerized legal information retrieval system, the classification of relevant data content and features is achieved, and its scalability is ensured to a certain extent. In this study, two algorithms are used: the image segmentation-based image information retrieval and the cuckoo algorithm-based content retrieval, and the two methods are combined to achieve the legal information retrieval function.

## 2. Algorithm and Design

### 2.1. Legal Image and Content Information Retrieval Algorithm

For legal images, the process of retrieval is a very important aspect, and it involves a large number of topics that are relevant and have certain regularity. Therefore, a system is needed to accomplish this task, i.e., to extract the data features contained in the images by means of computer technology, then to process these raw image data using algorithms, and then to store them in a database in text form as a searchable table of document space information according to the needs of the user.

Commonly used image features include color features, texture features, and shape features. At present, the mainstream color feature extraction algorithm mainly includes the following: color moment and color correlation map; the mainstream texture feature extraction algorithm mainly includes the following: gray symbiotic matrix, LBP texture, Tamura texture, wavelet transform method; the mainstream shape feature extraction method mainly includes the following: Fourier shape description method, shape invariant moment, and boundary feature description method. Finally, the extracted features are composed into a feature vector, which is used as the feature identity of this image in the CBIR system.

#### 2.1.1. Color Feature Extraction Method

The color moment is an important feature to represent the color of an object, and it is obtained by calculating the similarity between two images. In computer retrieval, we can use color to correlate with pixel values. It uses the mean value of image color (first-order moment), whose mean value is calculated as shown in equation (1), the variance (second-order moment), whose variance is calculated as shown in equation (2), and the skewness (third-order moment) to represent the image color feature, whose skewness is calculated as shown in equation (3).

(1) μ=i1N∑j=1NPi,j,

(2) $$\begin{array}{c} \sigma_{i}={\left\{\frac{1}{N}\sum_{j=1}^{N}{\left(P_{i,j}-\mu_{i}\right)}^{2}\right\}}^{1/2}, \end{array}$$

(3) $$\begin{array}{c} S_{i}={\left\{\frac{1}{N}\sum_{j=1}^{N}{\left(P_{i,j}-\mu_{i}\right)}^{3}\right\}}^{1/3}. \end{array}$$

After the third-order color moment of the image is calculated, a color moment feature vector is formed. Taking the HSV color space as an example, the color feature can finally be represented by the feature vector of

(4) $$\begin{array}{c} C=\left[\mu_{H},\sigma_{H},s_{H},\mu_{S},\sigma_{S},s_{S},\mu_{V},\sigma_{V},s_{V}\right]. \end{array}$$

The color correlation map is a representation of image color features, which reflects the spatial correlation of color with distance, and the color correlation map is defined as shown in equation (5). The color autocorrelation map can be expressed by equation (6).

(5) $$\begin{array}{c} \gamma_{C_{i},C_{j}}^{\left(k\right)}\left(I\right)=\underset{p_{1}\in I_{C_{i}},p_{1}\in I_{C_{i}}}{\mathrm{Pr}}\left[p_{2}\in I_{C_{j}}\left\|\right)p_{1}-p_{2}|=k\right], \end{array}$$

(6) $$\begin{array}{c} \alpha_{C_{i}}^{\left(k\right)}\left(I\right)=\gamma_{C_{i},C_{j}}^{\left(k\right)}\left(I\right)=\underset{p_{1}\in I_{C_{i}},p_{1}\in I}{\mathrm{Pr}}\left[p_{2}\in I_{C_{i}}\left\|\right)p_{1}-p_{2}|=k\right]. \end{array}$$

In a computerized legal information retrieval system, color features are a key aspect, that is, the realization of the query operation for the user. At present, traditional recognition techniques are used. This method has the following shortcomings:

1. The analysis and judgment of image color are not accurate enough, and the retrieval speed is slow. These problems lead to the inability of users to judge the characteristics of images, and a series of problems will occur in the later use process. Therefore, a machine learning method and neural network technology will be used to build, modify, and improve the database, and a computer language will be used as the development language to propose a set of procedures that can be applied to various image processing characteristic data, which provide users with fast and accurate access to legal information services.
2. Since the current laws and regulations do not provide for determination criteria to standardize the processing, it is impossible to determine the specific data type, which makes it difficult to effectively confirm the color classification results, thus causing the color search is not real and reliable. In order to enhance the real reliability of color retrieval, this study proposes a computer-based legal information retrieval system. It achieves color classification by processing different color images. The method can effectively reduce the color contrast value and brightness difference.
3. The color feature information is extracted in different locations to obtain whether there is a difference between the same or similar strings. However, since computer retrieval techniques are characterized by a certain degree of multitargeting and real time, biases can occur in their classification. The researchers proposed a classification method based on the text analysis technique (K-LY), first, an image processing software is used to generate feature vectors and convert them into numerical codes, second, the *k*-nearest neighbor algorithm is used to normalize the *k* character values, and then, a similarity metric with established similarity calculation rules is used to judge the number of characters that have been found to be different due to the large differences between the same strings.
4. The researchers proposed a classification method based on the text analysis technique (K-LY), first, an image processing software is used to generate feature vectors and convert them into numerical codes, second, the k-nearest neighbor algorithm is used to normalize the *k* character values, and then, a similarity metric with established similarity calculation rules is used to judge the number of characters that have been found to be different due to the large differences between the same strings. Researchers proposed a machine learning method and neural network based on the construction of a legal class digital resource retrieval model. The system is based on rules, it converts a large number of text images into text images with specific meaning (ID) through training and judgment, it realizes its feature extraction function, and it finally uses C# language to write programs to complete the system hardware and software design and development, so as to realize the operability, stability, and practicality of the legal information retrieval system.
5. The existing color feature extraction methods are slow and easily interfered with by other factors, which brings great inconvenience to people's life. In order to solve this problem, researchers have designed a hair information retrieval technique, which converts images into digital text form and stores them. This method largely overcomes the disadvantages and drawbacks of traditional recognition methods such as easy interference by noise, and at the same time, this can quickly and accurately extract the required content when there are a large number of uncertainties in data files such as pictures, text, and sound.
6. The existing color feature extraction methods have low recognition rates and cannot meet the practical needs of retrieval systems. A method based on “digital image processing techniques” has been proposed for this problem. The algorithm is to generate a data dictionary by converting the image into a comparison between different regions to obtain the required feature space values and then use the established interrelationship between the same characters in the image and text files to determine all the attribute information contained in the word, to derive the result set, and finally to retrieve and identify the results, so as to achieve the purpose of the legal query system.

#### 2.1.2. Texture Feature Extraction Method

**Figure 1:**
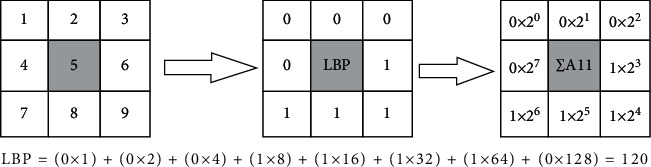
Calculation method of LBP eigenvalue.

The LBP features are the core of the system, which is mainly used to analyze all data types, information content, and attributes in the database. For a computer search user, the key issue is how to identify what the user has entered into the database that is associated with that query object yet can be distinguished. The calculation method of the basic LBP feature value is shown in Figure 1.

Ontological features of computers are used to classify and recognize image retrieval systems, that is, to extract the same attributes on different types of pictures. For the same figure, since its grayscale values, texture structure, and other information vary to some extent, the Tamura technique can be used to achieve similarity metrics and discrimination functions, and it also has the advantages of good scalability and ease of storage and processing [8].

Tamura is a text analysis-based retrieval method proposed in 1996, which is a technique that transforms datasets in different arrangements and uses specific words to express the intrinsic connection with all documents in the database. A computerized retrieval system can be used to achieve effective extraction, storage, and processing of a large amount of document information and other knowledge information and other resources, thus achieving effective knowledge retrieval and forming a new, efficient legal information management model with computer technology.

In terms of roughness, it is calculated as shown in equations (7)–(9).

(7) $$\begin{array}{c} A_{k}\left(x,y\right)=\sum_{i=x-2^{k-1}}^{x+2^{k-1}-1}\sum_{j=x-2^{k-1}}^{y+2^{k-1}-1}{\frac{g\left(i,j\right)}{2}}^{2k}, \end{array}$$

(8) $$\begin{array}{c} \left\{\begin{array}{c} E_{k,h}\left(x,y\right)=\left|A_{k}\left(x+2^{k-1},y\right)-A_{k}\left(x-2^{k-1},y\right)\right|,\\ E_{k,y}\left(x,y\right)=\left|A_{k}\left(x,y+2^{k-1}\right)-A_{k}\left(y,x-2^{k-1}\right)\right|, \end{array}\right) \end{array}$$

(9) $$\begin{array}{c} F_{\text{crs}}=\frac{1}{M\times N}\sum_{i=1}^{M}\sum_{j=1}^{N}S_{\text{best}}\left(i,j\right). \end{array}$$

In terms of contrast, it is calculated as as follows:

(10) $$\begin{array}{c} F_{\text{con}}=\frac{\sigma }{{\left(\alpha_{4}\right)}^{n}},\\ \alpha_{4}=\frac{\mu_{4}}{\sigma^{4}}. \end{array}$$

In terms of directionality, it is calculated as as follows:

(11) $$\begin{array}{c} \Delta G=\frac{\left(\left|\Delta_{H}\right|+\left|\Delta_{V}\right|\right)}{2}, \end{array}$$

(12) $$\begin{array}{c} \theta =\;\tan^{-1}\left(\frac{\Delta_{V}}{\Delta_{H}}\right)+\frac{\pi }{2}. \end{array}$$

Among them, the difference between the horizontal variation and vertical variation of the gradient vector is shown as follows:

(13) $$\begin{array}{c} H_{D}\left(k\right)=\frac{N_{\theta }\left(k\right)}{\sum_{i=0}^{n-1}N_{\theta }\left(i\right)}, \end{array}$$

(14) $$\begin{array}{c} F_{\text{dir}}=1-rn_{p}\sum_{p}^{n_{p}}\sum_{\varphi \in W_{p}}{\left(\varphi -\varphi_{p}\right)}^{2}H_{D}\left(\varphi \right). \end{array}$$

Although texture feature is an abstract image processing technique, it is also relatively mature, it has many shortcomings.

1. The accuracy in computer retrieval is not high. Because users have different needs for different types of images, there are often large differences between layers in different time periods, and image quality and storage space, etc., which all lead to a lack of data structuring, resulting in large errors when retrieval. A computer-based legal information retrieval system can be used to address common problems when searching by computer. It is based on digital graphs and proposes a new machine learning-based approach after analyzing and organizing existing technologies. The method is based on the combination of traditional databases and modern search engines to achieve structured data storage and query functions (including textual content), and the use of “automatic” search rules to improve its accuracy and efficiency.
2. Due to the limited capacity of database memory, the query and retrieval functions of the database are limited, resulting in a large number of deficiencies in the management of legal information resources. In view of the current situation of the application of computer technology in the judicial field, this study proposes a design idea of a computer-based legal knowledge retrieval system with the main purpose of classifying and integrating data. The method will be based on digital image processing (DCT) and other related algorithms to transform the original image into a format that meets the characteristic conditions and rules, and generate a simple query scheme by analyzing these rules, while also using its fast and convenient characteristics to meet user needs and enable the retrieval of legal information resources.
3. For texture attribute data, there are not enough sample points for effective utilization. Therefore, interconnection and analysis between databases are not possible. In computer search, it is necessary to use the rule extraction technology to classify the data in the database, and it can also extract information related to the text content by the existing rules. In this way, it can realize the needs of users, achieve the correlation between data, and also provide effective information for the system.
4. The quality of legal information retrieval can also be affected by aspects such as the lack of a regularized feature base and technical support, which can affect the final results. In order to solve this problem, this study proposes a method based on a computer technology feature library with technical support, which can transform the complex relationship processing in traditional entity law into a regularized logical reasoning process. When classifying the data, some special algorithms are also used to achieve interrelatedness among its attributes, and a legal information retrieval system for different user groups' features is designed by analyzing the functional requirements of the program, and it is used in combination with computer technology to improve the quality of legal information retrieval.
5. Texture feature extraction methods may have mismatches or omissions in the actual application, leading to unsatisfactory retrieval results. The researchers proposed a computer-based legal information retrieval method, which detailed the concept, features, and functions of the search engine and other knowledge points, and described it by analyzing the relevant rules and theories; second, MATLAB software was used to build a program implementation library and write source code for the program to complete the operational flow design and compilation process control test work, and finally, the whole result was displayed at runtime using C# language, which also provided some help for the legal information retrieval system.

The advantages of the texture feature extraction method are as follows: first, high adaptability to the process of extracting texture features from the original image before doing further analysis during computer retrieval. The method can transform the isolated, cluttered, and redundant information in the original blocks into a structured dataset with less regularity and global adaptability while also avoiding unnecessary losses caused by problems such as slow computation due to large space. Second, scalability, for existing computer retrieval systems, texture feature extraction must ensure that the image is analyzed and processed within a certain range before reaching a conclusion.

#### 2.1.3. Shape Feature Extraction Method

For computer retrieval systems, shape feature extraction is the key. This is because the process of compressing and analyzing images usually requires processing a large amount of raw image data, which are composed of many small-area objects. Within this large area, a simple and clear method can be used to obtain feature information and describe objects or other attributes that may also produce a set of distinctive characteristics and can be distinguished from each other, and the common method is the Fourier shape description method.

Fourier shape description is a kind of computerized legal information retrieval system, whose main function is to classify data containing various types of properties and characteristics of different attributes or characteristics of low structure or different speed of change. In the analysis of a large number of raw databases, a more correct, fast, and accurate conclusion can be obtained if a simple method is used.

The shape feature extraction method is based on the user's understanding of the image in the retrieval process, combined with graphic and attribute information for analysis and judgment; however, this method cannot determine the target area. Due to the limited computing power of the computer, only simple and fast and effective access to the two-dimensional image text of the image is generally selected as the retrieval object, and at the same time, the image processing process requires a large amount of data support and the difficulty of computer algorithm implementation, which leads to the general only feature extraction. In response to this problem, the design idea of a computerized legal information retrieval system is adopted, and the method of image processing as the key module and the database-based SQL Server model as the auxiliary function is selected after analyzing and comparing the existing technologies. The main advantage of this method is that the retrieval system is fully functional, easy to implement, simple to operate, and highly portable.

### 2.2. Content Retrieval Algorithm

The content retrieval algorithm used is mainly the cuckoo algorithm, which is mainly based on the principle of cuckoo flight. When the cuckoo bird flies in Levy, its step length satisfies the levy distribution, i.e., the step length *s* and its probability *L*(*s*) obey the Levy distribution, as shown in the following equation:

(15) $$\begin{array}{c} L\left(S;\mu ,c\right)=\sqrt{\frac{c}{2\pi }}\frac{e^{-c/2\left(s-\mu \right)}}{{\left(s-\mu \right)}^{3/2}}. \end{array}$$

Equation (15) is usually simply written as follows:

(16) $$\begin{array}{c} L\left(s\right)∼{\left|s\right|}^{-1-\lambda }\left(0<\lambda \leq 2\right). \end{array}$$

The nest finding path and location update formula for cuckoo search are shown as follows:

(17) $$\begin{array}{c} x_{i}^{\left(t+1\right)}=x_{i}^{\left(t\right)}+\alpha ⊕L\left(\lambda \right). \end{array}$$

In the actual calculation, the formula for the flight step is as follows:

(18) $$\begin{array}{c} s=\frac{u}{{\left|V\right|}^{1/\beta }}. \end{array}$$

*u* obeys a normal distribution with standard deviation *σ*_*u*_. *σ*_*u*_ is calculated as follows:

(19) $$\begin{array}{c} \sigma ={\left\{\frac{\Gamma \left(1+\beta \right)\times \;\sin\left(\pi \times \beta /2\right)}{\Gamma \left[\left(1+\beta \right)/2\right]\times \beta \times 2^{\left(\beta -1\right)/2}}\right\}}^{1/\beta }. \end{array}$$

According to equations (18) and (19), the cuckoo position update formula is shown in equation (20). Equation (21) is used to control the flight step [9].

(20) $$\begin{array}{c} x_{i}^{\left(t+1\right)}=x_{i}^{\left(t\right)}+\alpha ⊕\frac{u}{{\left|v\right|}^{1/\beta }}, \end{array}$$

(21) $$\begin{array}{c} x_{i}^{\left(t+1\right)}=x_{i}^{\left(t\right)}+\alpha ⊕\frac{u}{{\left|v\right|}^{1/\beta }}⊕\left(x_{i}^{\left(t\right)}-x_{\text{best}}^{\left(t\right)}\right). \end{array}$$

### 2.3. Design of Legal Information Retrieval System

**Figure 2:**
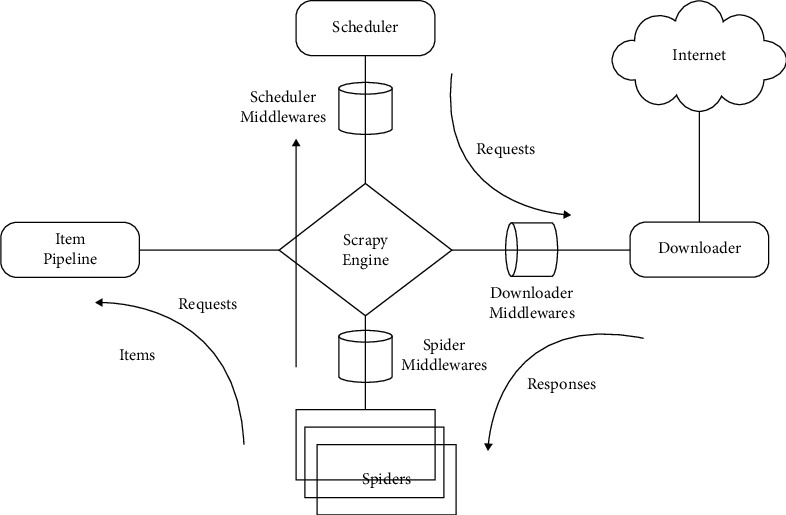
Scrapy framework.

The Scrapy framework is the core framework of the computer retrieval system, which contains the information exchange between users and technical service providers. Through this framework, users can search for various technical information, confirm the search results, and finally realize the query. The Scrapy framework is shown in Figure 2.

**Figure 3:**
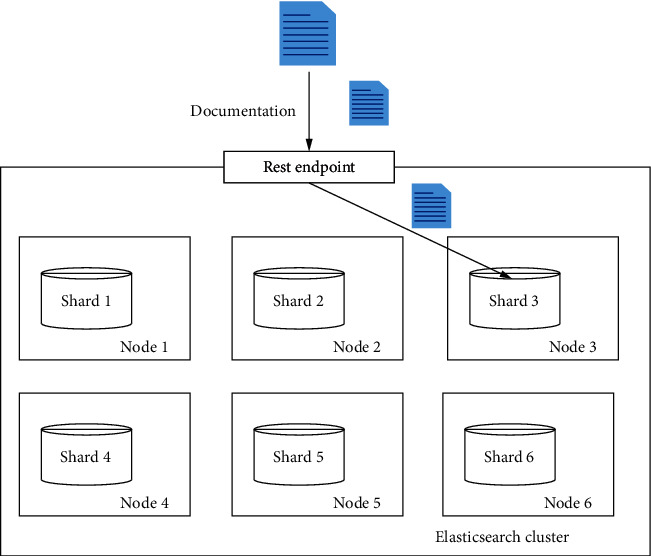
The process of building indexes in Elasticsearch.

This study focuses on indexing and querying information using Elasticsearch. The process of indexing is to analyze all the information in the retrieval system, draw conclusions, categorize it on this basis, build a complete database, and then use elastic classification technology to implement it. The first thing to do is to determine the object and subject matter to be searched and related attributes such as keywords, keyphrases, and other characteristic items; then, it obtains the original dataset information through regularized query commands and finally distinguishes all fields in the retrieval system that are related to the entity and have the same nature or category name according to a specific indexing method, thus forming a complete legal information retrieval system for the user's convenience [10]. The process of indexing by Elasticsearch is shown in Figure 3.

**Figure 4:**
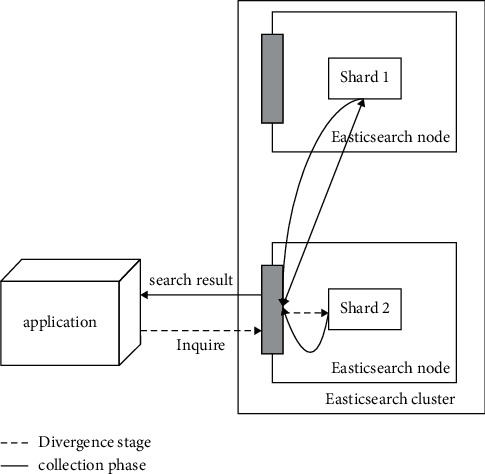
Elasticsearch executes the query.

In a computerized retrieval system, data query is very important because it directly affects the user's understanding and search of information content. Therefore, we want to implement a system that is functional, easy to use, and scalable. When the database needs to be searched, users must first enter the corresponding keywords to get the required results and return them to the main page, so as to meet the demand, and if there is no corresponding statement that cannot get all the characteristics of the retrieved data containing keywords or attributes, then users can also click the “query” button or other auxiliary methods to complete the search process [11]. The process of query execution by Elasticsearch is shown in Figure 4.

**Figure 5:**
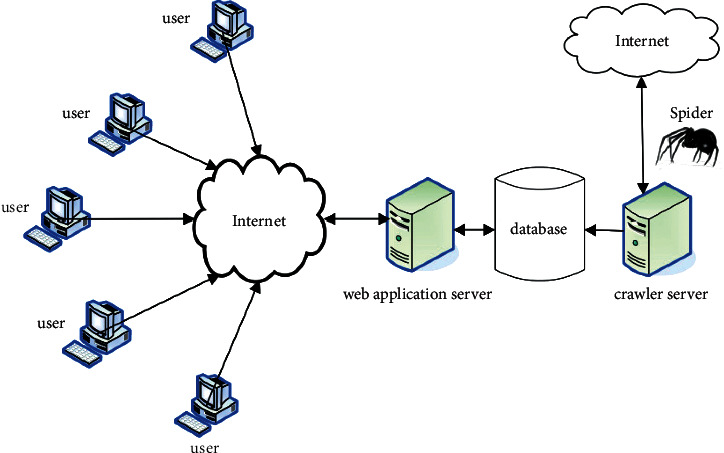
System network environment.

The computer operating environment is mainly built by using a large platform to divide the user management, retrieval rules, and system structure, and then provide corresponding function modules according to different types of information to realize the user's retrieval, and the system network environment is shown in Figure 5. The design of the system is mainly to study the computerized legal information retrieval system. In this process, the relevant personnel first need to understand the development of computer technology and the existing relevant laws and regulations in China, and so on. Second, it analyzes and compares related network management, infringement processing, and other aspects of information protection methods and procedures at home and abroad, then formulates the corresponding functional module diagram structure according to different types of problems, and finally uses the Java language to realize a digital image-based evidence transformation.

### 2.4. Functional Design of the System

**Figure 6:**
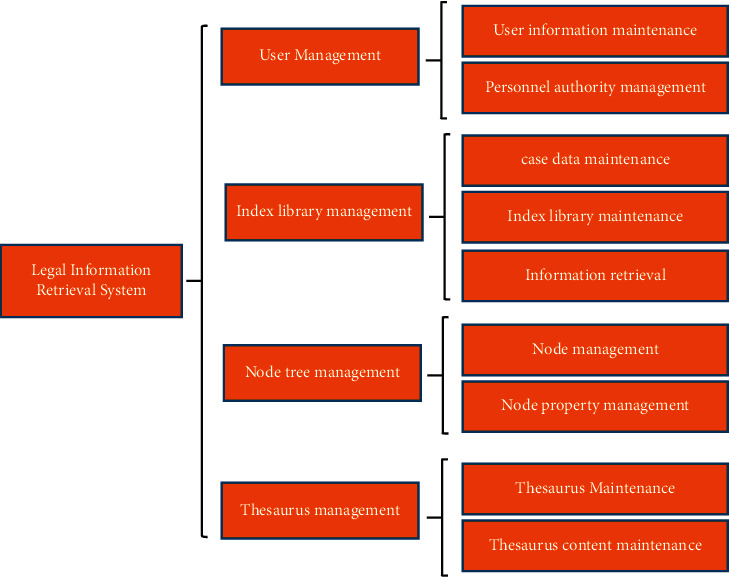
System functional design.

The computer retrieval system is designed to solve the problems encountered in real life, and its main functions are shown in Figure 6.

1. User management. The system can realize the addition, deletion, modification, and checking of user information. Different operation methods are set for people with different authority, so as to ensure that they can operate the corresponding search function; in addition, the query results are recorded in the database and saved to the database before returning to the next user.
2. Index library management. Index library is an important part of the retrieval system, through the analysis of computer functions that will be stored in the database according to certain rules in an orderly manner. For a query site, the key is to have data sources and indexes. If there is no such need, there will not be any problem with the content of the page or the level of interest of the user on the page, and when we have set up all the sites, then the whole program can normally run, so it is necessary and very important to study the legal information retrieval system of the computer [12].
3. Node tree management. The node tree is a method of classifying the abstract entity elements by putting them into categories, which can be used in data mining and can find the corresponding type of documents in a particular scenario. When an object is selected, its attribute information needs to be managed. First, a rule tree is built and feature extraction techniques are determined based on the object, and then, the tag set of the target character set (candidate nodes) is generated after converting the identification text into a string block, i.e., the tagged entities, so that the path length of the content contained in the target text and the corresponding data stream size can be obtained. Finally, the label set of candidate nodes is compared with the target character set by the rule tree classifier to obtain relevant information.
4. Thesaurus management. Thesaurus management is one of the important functions of the retrieval system, which is mainly to analyze all the included data in the database and then generate the corresponding tables. The methods used in the process are as follows: first, the database contains query conditions or other relevant information and is added to the “table,” and second, the data feature values and attribute values in the table are extracted by keywords. Among them, for the query conditions have been entered or there are errors in the record that should be deleted in time, so as to avoid affecting the accuracy and integrity of the search results, resulting in retrieval failure [13]. As for the entries that have been entered with query conditions or other related contents, they should be analyzed and tables should be generated according to the characteristics of the data information in the database.

## 3. Testing Results of the Legal Information Retrieval System

Testing is mainly for the design of the retrieval system, which can be tested in terms of function, performance, and security. This can ensure that the whole legal information retrieval system can normally operate. For users, they can query the relevant documents by logging in, but due to a large amount of data in the database and its complexity and variety, the users cannot directly use the technology to obtain the relevant content, and there is also the case that some keywords are maliciously altered or deleted by others. The amount of information in the database is small, and the query time is long. These problems cause the system to not operate normally. Therefore, when designing a computer legal information retrieval system, it is necessary to combine it with traditional technology, and then test and analyze the system.

Software testing is an important part of software development engineering, and it is the key to ensure the quality of software. The purpose of software testing is to find out the problems in software and correct them in time, so as to ensure the normal use and functional perfection of the system. In the process of testing, if no problem is found, it does not mean that the program is all right but only means that there is no problem in testing for the time being, so the software needs to be tested. In the process of developing the computer legal information retrieval system, there are many problems. For example, the method is not suitable for complex dataset processing and analysis and cannot meet the user needs, etc. Therefore, in order to improve the level of computer understanding of the relevant aspects of knowledge and the degree of perfection of the software function and the development of this type of system to solve these problems, adequate testing can ensure the perfect functionality of the system, improve the quality of legal information retrieval, and thus provide more complete, reliable, and timely services to users. The concept of software quality is a multidimensional concept, and we cannot state the quality of the system only in terms of one aspect of the system but need to consider the characteristics of the system in many aspects to specify the quality of the software system. This study uses testing methods mainly black-box testing, white-box testing, risk-based testing, and model-based testing.

### 3.1. Black-Box Testing

**Figure 7:**
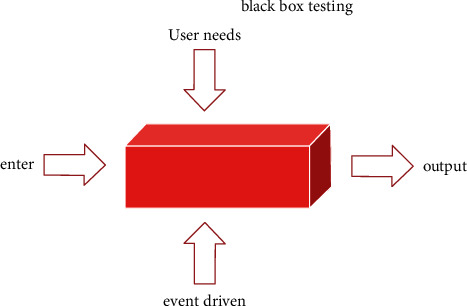
Black-box testing.

During the testing process, we can see that there are no problems or errors in the operations performed after the system functions are designed. In this way, we can avoid logical errors due to excessive input parameters, and we can also avoid operational errors due to excessive input parameters. Black-box testing is to detect whether there are bugs in the internal code of the software or to check the integrity and correctness of the data when it is running [14]. Black-box testing is an effective way to improve system performance, reduce risk, and improve software functionality. Black-box testing is shown in Figure 7.

### 3.2. White-Box Testing

It is also known as logic-driven design, and it is a method of checking the results of program operation and then determining whether the system is properly working. It mainly focuses on user input and output data and error situations during actual execution. It determines whether the program functions well by deriving data from the test system and evaluating the results. The purpose of white-box testing is to identify the logical relationships and physical connections between the various parts of the system, which ensures that the program has good performance and can be used in a reasonable manner [15].

### 3.3. Risk-Based Testing

In risk-based testing, the structure and functions of the retrieval system are determined according to the risks predicted in advance and then evaluated according to the priority of risk prediction, to complete the retrieval of computerized legal information resources.

### 3.4. Test Environment

The computer test environment is the laboratory where the actual operation can be carried out, including computers, electronic machines, and other electronic equipment. For the performance of the computer, it mainly depends on whether it meets the standards. If it does not meet the requirements, it will not be able to properly operate, and when these conditions are not available, software development work cannot be carried out to achieve the system functions. Therefore, the design process needs to take into account the impact of different aspects on the entire software project and then start testing to make sure that each part can properly operate before it is officially put into use.

### 3.5. Testing Process

The testing of this system is an assembly test and confirmation test of the completed software system, to be conducted for the entire product system, for testing whether the system meets the requirements specifications, to identify deficiencies, and to propose more perfect solutions. Our system testing mainly includes two parts: functional testing and robustness testing. Functional testing is mainly the functional testing of the system, i.e., the verification of the functions of each part to see whether the user requirements are met. Robustness testing is used to test the security, fault tolerance, and recovery capability of the software system, and to evaluate the speed of the system.

### 3.6. Test Results and Analysis

In practice, we use Oracle 10g as the basic information storage database and test the system one by one according to the functional requirements analyzed during the requirement analysis. During the testing process, the focus is on whether the nonfunctional requirements of the system meet the requirements. After the development of the system, the system was deployed and installed on the test machine, and the system was functionally operated. During the operation, the basic performance requirements of the system were achieved. The test results show that the computerized legal information retrieval technology is a very perfect and reliable database management method, and users can quickly and conveniently query relevant legal policies and documents and other information.

## 4. Conclusions

In conclusion, the development of this system implements a computer-based legal information retrieval system. First, the database is analyzed; then, rules are designed in the database, such as user name and password, query method (including text/speech), and data type. Finally, the program is written in C language and burned on top of the Microsoft Windows interface. The request is requested from the server through the browser, and the result is displayed so that it is easy for the operator to use. It also implements the functions of user registration, login, infringement, etc., and uses Java language to operate the database in the legal information retrieval system. At the same time, this function can be used to transform traditional paper documents into information resources that can be directly retrieved and saved, saving a lot of time and human and material costs. With the development of computers, people have more and more new needs for retrieving information. The traditional legal search system has many shortcomings, such as imperfect laws and regulations, no unified standards, and different ways, which seriously restrict the development of the retrieval system. In order to solve these problems, this study proposes a new system with the goal of designing a computerized legal information retrieval system, which can ensure the realization of functions while also making it easy for operators to grasp and use.

## Data Availability

The data used to support the findings of this study are available from the corresponding author upon request.

## References

[1] Kumar R., Sharma S. C. (2018). Information retrieval system. *International Journal of Technology Diffusion*.

[2] Kanapala A., Jannu S., Pamula R. (2019). Passage-based text summarization for legal information retrieval. *Arabian Journal for Science and Engineering*.

[3] van Opijnen M., Santos C. (2017). On the concept of relevance in legal information retrieval. *Artificial Intelligence and Law*.

[4] Wang B., Qian Y., Heled J., Yuan A. (2018). Image Retrieval Algorithm Based on Minimal Loss Hashing. *MATEC Web of Conferences*.

[5] Lu S., Wang B. (2019). An image retrieval algorithm based on improved color histogram. *Journal of Physics: Conference Series*.

[6] Deng J., Zhao L. (2015). Image Edge Detection Based on Improved Cuckoo Search Algorithm. *Computer System Applications*.

[7] Lipponen A., Mielonen T., Pitkänen M. R. A. (2018). Bayesian aerosol retrieval algorithm for MODIS AOD retrieval over land. *Atmospheric Measurement Techniques*.

[8] Ma W. (2020). Three-dimensional point cloud registration algorithm based on cuckoo optimization [J]. *Computer Applications and Software*.

[9] Zhang X., Wang X. (2018). Review of Cuckoo Search Algorithms. *Computer Engineering and Applications*.

[10] Song L. L., Wang Q. Hu, Pei Z. Li (2014). An Image Retrieval Algorithm Base on Texture Features. *Applied Mechanics and Materials*.

[11] Li Y., Liu S., Zhu P., Yu J., Li S. (2017). Extraction of visual texture features of seabed sediments using an SVDD approach. *Ocean Engineering*.

[12] Shi Y., Wang L. W., Lan H. Y., Zeng G. (2014). Extraction of the Palm Vein Texture Features Based on Gabor Wavelet Transforms. *Applied Mechanics and Materials*.

[13] Yazid H., Elouni F., Kalti K., Tlili K. (2011). Un système informatique d’archivage et d’indexation de cas d’IRM de tumeurs cérébrales. *Feuillets de Radiologie*.

[14] Groz R., Bremond N., Simao A., Oriat C. (2020). hW-inference: a heuristic approach to retrieve models through black box testing. *Journal of Systems and Software*.

[15] Mahapatra A., Malu R. (2013). Temporal white box testing using evolutionary algorithm. *International Journal of Innovative Research and Development*.

